# Ischemic stroke in young patients in Medellín, Colombia

**DOI:** 10.1186/s12883-022-02895-9

**Published:** 2022-09-22

**Authors:** Juan Diego Vargas-Murcia, Sandra Patricia Isaza-Jaramillo, Dionis Magnary Vallejo-Mesa, Daniela Carvajal-Muñoz

**Affiliations:** 1 Neurology Section, University of Antioquia, Carrera 51D #62-29, Medellín, 050010 Colombia; 2 School of Medicine, University of Antioquia, Medellín, Colombia

**Keywords:** Stroke, Young adult, Risk factors, Etiology, Colombia

## Abstract

**Background:**

There is scarce information about ischemic stroke in young patients in Colombia. To get insights about this phenomenon, this study describes the etiologies and risk factors of ischemic stroke in young patients in a third level complexity referral hospital in Medellin, Colombia.

**Methods:**

A retrospective observational cross-sectional study was carried out reviewing the medical records of patients between 18 to 49 years old admitted for the first time for ischemic stroke, from January 2009 to December 2019. The sociodemographic characteristics, risk factors, and etiological classification of ischemic stroke according to the Trial of Org 10,172 in Acute Stroke Treatment (TOAST) were described.

**Results:**

Two hundred thirty-seven cases were found. The most frequent risk factors were arterial hypertension (31.7%), smoking (29.5%) and alcohol intake (23.2%). There was a greater number of traditional cardiovascular risk factors at older ages. The TOAST classification was large-artery atherosclerosis (6.8%), cardioembolism (17.7%), small-vessel disease (7.6%), other determined etiology (25.7%) and undetermined (42.2%). Within cardioembolism, the most common high-risk source was valve replacement, and the most common moderate-risk source was patent foramen ovale. Craniocervical arterial dissection (11.4%) and substance abuse (2.9%) were the two most frequent sources within other determined etiologies. The most common compromised vascular territory was the anterior (55.7%).

**Conclusions:**

The high frequency of traditional risk factors in young patients highlights the need to optimize primary and secondary prevention plans. This study provides new insights about the relevance of illicit substance abuse in Colombia as a cause of stroke in young patients, unlike the previous one conducted in Bogotá. Infectious causes were other peculiarities found. It is necessary to investigate the reasons for the high proportion of undetermined causes.

**Supplementary Information:**

The online version contains supplementary material available at 10.1186/s12883-022-02895-9.

## Background

Strokes are the second cause of mortality worldwide and an important cause of disability [1]. Ischemic stroke in young patients has been defined with varying cutoff points; the most commonly used range is 18-49 years [2]. It corresponds to approximately 10% of all strokes [3].

The incidence of stroke in young patients has risen [4]. It presents a geographical variation from 18.1/100,000 persons per year in some European regions [5], to up to 100/100,000 persons per year in Sub-Saharan Africa [6]. A male predominance has been observed, although it is more common in women younger than 30 years [7, 8]. This difference is probably due to female sex specific risk factors such as oral contraceptives, pregnancy, or postpartum period [9].

Strokes in a younger population have a devastating impact on the patient’s personal, familial, and social lives. These patients experience higher survival probabilities and longer time living with disabilities than patients who suffer a stroke at an older age, they endure lower quality of life and higher burden of costs from healthcare systems [10, 11]. Risk factors and causes of stroke are different from those that are seen in elderly adults. Nonetheless, an increase of traditional vascular risk factors has been observed in younger patients [4]. An undetermined etiology is the most common one found in most studies, and the craniocervical arterial dissection is an important and specific cause [4].

There is scarce information from Colombia regarding strokes in the younger population, information gathered is mostly from case reports and case series [12]. The most important paper described the causes and risk factors in 152 young patients with ischemic stroke. Smoking history (19%), history of high blood pressure (18%), and presence of cardiovascular disease (17%) were the most frequent traditional risk factors. Etiological classification on the other hand was distributed as undetermined (33.5%), other determined etiology (33.5%), cardioembolism (23.6%), large-artery atherosclerosis (6.5%), and small-vessel disease (2.6%). None of the subjects had stroke secondary to substance abuse [13].

The objective of the present work was to describe the etiology and risk factors of ischemic stroke in young patients admitted for the first time to a tertiary university hospital from Medellín, Colombia, during 2009-2019, performed through chart review.

## Methods

This is a cross-sectional, observational study of young patients with ischemic stroke who were admitted over a 10-year period (2009-2019) to IPS Universitaria Clínica León XIII. Eligible patients were between 18 and 49 years old and had been diagnosed for the first time with ischemic stroke at this hospital. Patients with no ischemic stroke on neuroimaging were excluded. Patients with transitory ischemic attack (TIA), intracranial hemorrhage (ICH), subarachnoid hemorrhage (SAH), sinus vein thrombosis (SVT) with or without ischemia, ischemic stroke secondary to endovascular procedures (aortocervical, coronary, endarterectomy), head trauma, intracranial or cardiac surgery were also excluded. To do more accurate comparisons with some of the most important studies of ischemic stroke in young patients with similar methodology, TIA cases were excluded. Additionally, normal brain imaging results could include transient paroxysmal events that mimic TIA.

A battery of studies were used to establish “undetermined cause”; these included a complete blood count, basic metabolic panel, and antiphospholipid antibodies (anticardiolipin antibodies, anti-beta2 glycoprotein 1 antibodies, and lupus anticoagulant). Selected studies such as hypercoagulable panel tests (protein C, protein S, and antithrombin III levels, prothrombin gene mutation, factor V Leiden mutation), hematologic panel tests (serum protein electrophoresis, homocysteine level), serum and urine toxicology screen, and others were performed on a case-by-case basis. These results were not available in all cases.

Cardioembolic sources of stroke were identified by transthoracic echocardiography or transesophageal echocardiography in uncertain cases. All patients underwent electrocardiogram and some cases Holter monitoring. The main brain imaging used to typify TOAST were magnetic resonance angiography (MRA) reports evaluated by neuroradiologists.

The data source was medical records. Data obtained included demographic characteristics, comorbidities, family history, stroke etiology, and first laboratory results, with absolute and relative frequencies. Age was grouped as median and interquartile range given that the distribution was non-normal according to the Shapiro-Wilk test. Data was collected on an excel spreadsheet and was analyzed through SPSS 25 software. As TOAST classification [14] was essential, investigators who collected data from medical records had to take a training session and a written test to prove an excellent comprehension of the material.

This study was performed with prior endorsement by the ethics committee—according to the Helsinki declaration—and the international ethics guidelines for healthcare research on human beings—performed by the council for international organizations of medical sciences (CIOMS) and world health organization (WHO) [15]—so data confidentiality was preserved.

## Results

Two hundred thirty-seven subjects had complete data. Most patients were women 53.2% (126 subjects), with 40 years of age (median 40 years; interquartile range 12; Q_1_ 34 – Q_3_ 46). The complete description of the characteristics of the subjects, including the frequency of risk factors and stroke etiologies are presented in Table 1. Demographic characteristics and risk factors according to sex and age are presented in Supplementary Table 1.

**Table 1:** Characteristics of the subjects

Characteristic		Descriptive statistic (*N* = 237)
	Absolute frequency	Relative frequency (%)
Obesity	30	12.7
Dyslipidemia	20	8.5
Smoking	70	29.5
Arterial hypertension	75	31.7
Diabetes	19	8.1
Without Sleep apnea	234	98.7
Cancer	7	3.0
HIV	2	0.8
Syphilis	3	1.3
Alcohol intake	55	23.2
Marihuana	21	8.9
Cocaine	23	9.7
Other substances	8	3.4
Use of oral contraceptives	9	3.8
Postpartum	2	0.8
Ischemic cardiac disease	7	3.0
Heart failure	14	5.9
Peripheral arterial disease	3	1.3
Previous ischemic stroke	30	12.6
Family history of ischemic stroke	20	8.4
Valvular AF	2	0.8
Non valvular AF	1	0.4
Valve replacement	12	5.0
Without PFO	233	98.3
Without ASA	233	98.3
Low levels of Vitamin B12	27	11.4
Migraine	22	9.2
APS	2	0.8
SLE	8	3.4
Anticardiolipin antibodies	17	7.2
Lupus anticoagulant	18	7.6
PC deficiency	3	1.3
PS deficiency	7	3.0
Hyperhomocysteinemia	4	1.7
Factor V Leiden	1	0.4
Without Prothrombin deficiency	7	3.0
Other thrombophilia (AT-III deficiency)	1	0.4
**Stroke etiology**		
Craniocervical arterial dissection	28	11.8
Large-artery atherosclerosis	17	7.2
Small-vessel disease	16	6.8
Substance abuse	11	4.6
PFO	11	4.6
Valve replacement	11	4.6
Dilated cardiomyopathy (ejection fraction < 35%)	10	4.2
Atrial fibrillation	8	3.4
Intracardiac thrombi	6	2.5
APS	5	2.1
Rheumatic cardiac disease	4	1.7
Central nervous system primary vasculitis	3	1.3
Infectious endocarditis	3	1.3
PFO + ASA	3	1.3
Moyamoya syndrome	3	1.3
Reversible cerebral vasoconstriction syndrome	3	1.3
Left ventricle hypokinesis	2	0.8
Tuberculous vasculitis	2	0.8
Meningovascular syphilis	2	0.8
CADASIL	1	0.4
Fibromuscular dysplasia	1	0.4
Hypercoagulability secondary to HIV	1	0.4
Hypercoagulability secondary to nephrotic syndrome (primary glomerulonephritis)	1	0.4
Hypercoagulability secondary to nephrotic syndrome (Diabetic nephropathy)	1	0.4
Hypoperfusion post cardiac arrest post	1	0.4
Acute myocardial infarction	1	0.4
Heart failure	1	0.4
Irregularity of intracerebral vessels	1	0.4
Paraneoplastic hypercoagulability syndrome	1	0.4
Thrombophilia (protein S deficiency)	1	0.4
Vasculitis	1	0.4
Acquired thrombophilia (protein S deficiency, protein C and ATIII)	1	0.4
SLE vasculitis	1	0.4
Toxic vasculitis secondary to cocaine abuse	1	0.4

*AF* Atrial fibrillation, *APS* Antiphospholipid syndrome, *ASA* Atrial septal aneurysm, *AT-III* Antithrombin III, *HIV* Human immunodeficiency virus, *PC*: protein C, *PFO* Patent foramen ovale, *PS* Protein S, *SLE* Systemic lupus erythematosus

In 26.2% (62) no data about education was found, 7.6% (18) reported no education, 42.6% (101) coursed elementary school, 8.0% (19) high school, and 15.6% (37) completed undergraduate education.

80.2% (190) of subjects came from urban areas. 37.6% (89) were single, 33.3% (79) were married, 25.7% (61) were in consensual union, widows and divorcees 1.2% (3) and no data available in 2.1% (5).

The anterior vascular territory prevailed 55.7% (132) followed by the posterior 30.0% (71) and multiple territory 14.3% (34). TOAST classification in large-artery aterosclerosis 6.8% (16), cardioembolism 17.7% (42); Small-vessel disease 7.6% (18); other determined etiology 25.7% (61) and undetermined 42.2% (100).

Cardioembolism was one of the causes described. On Table 2, the cardioembolic cause is specified for the 42 subjects who presented with it. The most common high-risk source was valve replacement, and moderate risk was patent foramen ovale.

**Table 2:** Frequency of cardioembolic causes (*n* = 42)

High risk sources	N^a^	%
Dilated cardiomyopathy (ejection fraction < 35%)	9	21.43
Intracardiac thrombi	6	14.29
Infectious endocarditis	2	4.76
Rheumatic cardiac disease	4	9.52
Atrial fibrillation	6	14.29
Acute myocardial infarction	1	2.38
Valve replacement	10	23.81
Moderate risk sources	N	%
Left ventricle hypokinesis	2	4.76
Heart failure	1	2.38
Patent foramen ovale	8	19.05
Patent foramen ovale + atrial septal aneurysm	3	7.14

^a^ The number of causes is higher than the total number of subjects due to some subjects having more than one cardioembolic source

According to TOAST, patients were classified as undetermined if no cause was found despite extensive work-up. This includes patients who had 2 potential causes, or those in whom work-up couldn’t be completed. Cause was established in a majority of the subjects; this wasn’t achieved in 39.2% (93). Table 3 describes the cause for those subjects with a single cause.

**Table 3:** Frequency of causes classified as other determined causes (*n* = 61)

	N	%
Substance abuse	7	11.48
Craniocervical arterial dissection	27	44.26
Fibromuscular dysplasia	1	1.64
Hypercoagulability secondary to nephrotic syndrome (primary glomerulonephritis)	1	1.64
Hypercoagulability secondary to nephrotic syndrome (Diabetic nephropathy)	1	1.64
Hypoperfusion post cardiac arrest post	1	1.64
Meningovascular syphilis	2	3.28
APS	5	8.20
Paraneoplastic hypercoagulability syndrome	1	1.64
Moyamoya syndrome	3	4.92
Reversible cerebral vasoconstriction syndrome	2	3.28
Thrombophilia (protein S deficiency)	1	1.64
Acquired thrombophilia (protein S deficiency, protein C and ATIII)	1	1.64
Vasculitis	1	1.64
SLE vasculitis	1	1.64
Central nervous system primary vasculitis	3	4.92
Toxic vasculitis secondary to cocaine abuse	1	1.64
Tuberculous vasculitis	2	3.28

*APS* Antiphospholipid syndrome

*AT-III* Antithrombin III

*SLE* Systemic lupus erythematosus

**Fig. 1:**
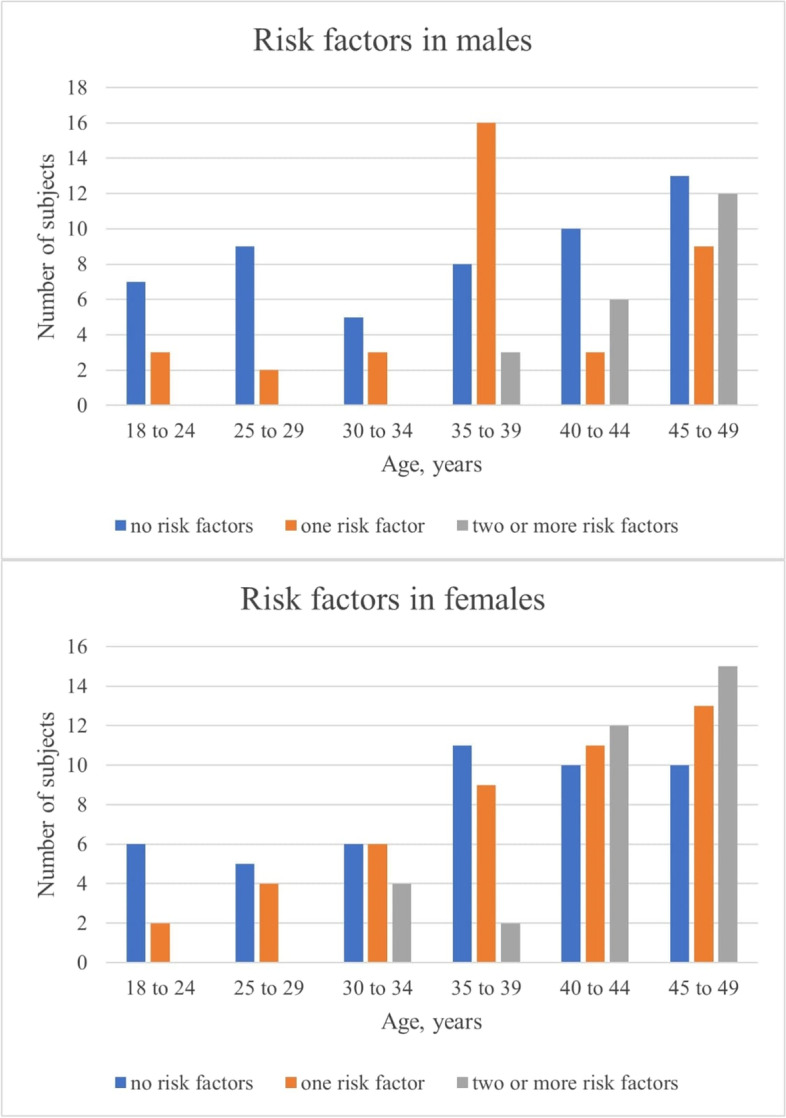
Traditional cardiovascular risk factors by sex and age group. Traditional cardiovascular risk factors: Obesity, dyslipidemia, arterial hypertension, diabetes, smoking. * Modified from: Maaijwee NA, et al. Ischaemic stroke in young adults: risk factors and long-term consequences. Nature Reviews Neurology. 2018 [16]

**Fig. 2:**
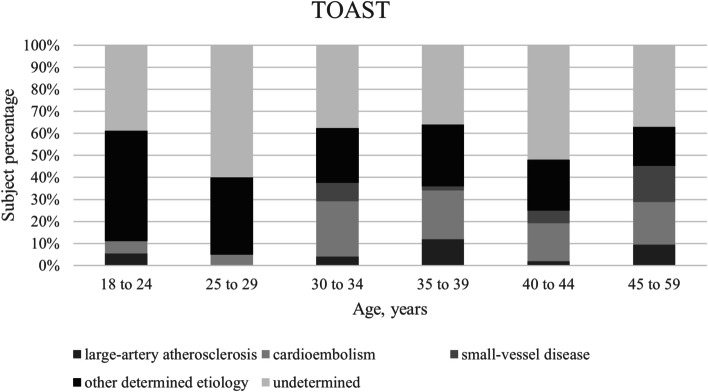
TOAST category by age group. * Modified from: Putaala J, et al. Analysis of 1008 Consecutive Patients Aged 15 to 49 With First-Ever Ischemic Stroke. The Helsinki Young Stroke Registry. Stroke. 2009 [7]

The tendencies of the number of risk factors by sex and TOAST distribution for different age groups are charted on Fig. 1 and Fig. 2, respectively. A higher number of cardiovascular risk factors was observed at higher ages. In younger age groups, the prevailing TOAST categories were “other determined causes” and “undeterminate”; meanwhile, those at higher ages had an increase on the categories “large-artery atherosclerosis”, “small-vessel disease” and “cardioembolism”.

## Discussion

This descriptive study of ischemic stroke in young patients is the biggest one done in Colombia so far. It was performed in a University Hospital. Most patients were female, concordant with most recent global data [4], but it’s differs to previous case series [3, 7, 10, 17–24].

In the literature predominance of female over males under 30 years was observed [7, 8, 21, 23, 25],, but in this study was the opposite which is similar to other studies [26]. However in this study, over 30 years, female predominance was observed, contrary to most series. A recent cohort study found a higher ischemic stroke incidence on female patients of all age groups, particularly between 18 and 44 years [27]; this merits further research, and could be explained by an improvement on healthcare access, biases on previous descriptive studies, among other things.

A high frequency of traditional risk factors, specially arterial hypertension and smoking, was observed, which is similar to the evidence previously published [3, 7, 8, 10, 13, 17, 18, 20–22, 24, 26, 28–34]. Nonetheless, dyslipidemia wasn’t identified as one of the main risk factors, unlike previous studies [3, 7, 8, 10, 17, 21, 22, 24, 26, 29, 31–33]. In some series, alcohol consumption has been described as one of the main risk factors [20–22, 25, 32], similar to what was observed in our study. The age-related increase of such traditional risk factors may explain, in part, a similar tendency on the atherosclerotic and lacunar stroke; nevertheless, the influence of these traditional risk factors is unclear for the larger proportion of patients with undetermined etiology and other determined etiologies observed at younger age.

A remarkable finding in the present study is the high rate of illicit substance consumption, compared to previous studies [7, 13, 21, 26], which is directly related to the determined etiologies of stroke in this series. The rate of substance abuse-related stroke (2.9%) is high compared to European series which have reported a 0.3-1.5% rate [7, 20, 26, 35] which is double or even 10 times the case number. Additionally, substance abuse participated on the genesis of the vascular event with other causes, hence being classified as undetermined etiology, but it remarks the importance of this matter. In general, the most commonly implied substance was cocaine-induced vasculopathy. In Latin-american case series, this cause was rare [13, 19, 25].

The frequency of migraine was discretely lower than the general population [36], which differs from previous studies that consider migraine an important risk factor, primarily on women [7, 13, 24, 26, 28, 33, 37]. The low frequency may be explained by underdiagnosis, and/or a lower relevance on this population. Other possible explanations may be considered. Most studies found a low rate of migrainous infarction on 0.2-2.8% [3, 7, 13, 19, 24, 26, 28, 29, 31, 33, 35] while other studies found a slightly higher rate (3.3-4.8%) [20, 25]. The present study found no migrainous infarction cases similar to an Italian case series [8].

Unlike previous reports, with high frequency of oral contraceptives use on younger women which could explain a female predominance at younger ages [3, 7, 20, 21, 24, 26, 28, 33, 34], this wasn’t observed in the present study.

The results according to TOAST classification from this study are similar to those obtained on the largest series (to our knowledge) which included 3331 patients in multiple centers from Europe [35]. The high frequency of undetermined TOAST in the present study may be explained by various factors. Nonetheless, some patients underwent different diagnostic studies at different institutions, hence, the result was unknown; in other cases, it wasn’t possible to access the chart data or diagnostic studies results in older charts, which made it difficult to determine possible etiologies of stroke.

The proportion of large-vessel atherosclerosis (6.8%) was similar to previously published series in Europe and South America (6.7-9.3%) [7, 19, 21, 26, 35], and particularly to the previous Colombian study (6.5%) [13]; Mexico has reported lower rates [25].

Small-vessel disease in this study (7.6%) was lower than what was previously reported (12.2-42.5%) [7, 19, 21, 22, 26, 29, 31, 35], however, it triples the previous Colombian study which found a 2.6% rate [13]. It was similar to the Italian and Swiss series (5-9%) [3, 20]. A subject was diagnosed with CADASIL, a condition of interest in our country [37, 38].

The frequency of cardioembolic etiologies (17.7%) was similar to the European publications (15.8-20.1%) [7, 20, 21, 26, 29, 35], although lower than previous reports in Latin-America (23.6-28.3%) [13, 19, 25]. It is remarkable the higher frequency of rheumatic heart disease in our study (10%) and other developing countries [19, 25] compared to European countries [7, 20]. On the other hand, the frequency of cardioembolism was higher in the present study compared to a french one [17], probably due to low-moderate risk cardioembolic sources were classified as undetermined causes.

Cardioembolic rates were higher at older ages, mostly over 30 years, as has been previously described [8, 19, 24, 25, 33].

The rate of other determined etiologies (25.7%) was similar to those reported by European case series between 19 and 29% [3, 7, 8, 17, 20, 21, 24, 26, 28, 29, 31, 35]. Conversely, Latin American case series have reported a higher rate of this category, between 33.5-39.6% [13, 19, 25]. The most common determined etiology was craniocervical arterial dissection, in 11.4% of cases, similar to the 12.8% found in the largest study [35], and in the same direction as previous studies which report values up to 24% [3, 7, 8, 13, 17, 24–26, 28, 31].

It’s worthwhile mentioning that a vasculitis case of unconfirmed etiology was observed, although with high suspicion for neurocysticercosis. This etiology has been observed in Brazil and Mexico case series, with frequencies of 1.9 and 4.6%, respectively [19, 25]. A Colombian case of ischemic stroke due to neurocysticercosis related vasculitis has been reported [39]. Another remarkable findings was two cases of tuberculous vasculitis in our series, an etiology not reported in previous series; hence, the proposal of considering it as a potential etiology in undetermined cases, particularly in countries where *Mycobacterium tuberculosis* infection is endemic. Additionally, meningovascular syphilis was another neuro-infection causing stroke.

Patients with more than one potential etiology of stroke, and those with no etiology identified despite extensive/insufficient work-up, were included in the undetermined etiology category. This category obtained the higher proportion in our study (42.4%), similar to previous studies (32-44%) [3, 7, 20, 21, 25, 29, 35, 40]. One of the previous series presented a higher rate (62.4%) of undetermined cases [17], which may be explained due to potential cardioembolic sources (such as PFO or ASA) being classified as undetermined.

The vascular territory was determined with neuroimaging results. The most commonly involved was the anterior one, similar to most previous studies [3, 7, 8, 13, 18, 20, 22–24, 26, 32, 34]. The distribution of involved territories was very similar to previous Colombian series [13]. Despite the frequent use of cerebral MRA, no high rate of posterior circulation involvement was observed unlike some previous studies [7, 24, 26].

Among the strengths of the present study include that it was performed on a University Hospital which takes care of a great volume of patients from all economic strata, which could potentially be reflected on the wide variety of etiologies. The biggest series on Colombia and the world were developed on University Hospital, just as the present study. However, a larger number of determined etiologies was observed, compared to the previous Colombian series, despite being developed in similar times [13]. Another strength was the description of demographic characteristics, which may support further studies to explore social determinants of health in these patients.

There are some limitations in our study. One being the high frequency of the undetermined etiology as was previously developed. The observational design has intrinsic biases. Variable definition depending on chart report may be different from previous studies, which implies some difficulties for comparison. On substance consumption, it wasn’t possible to determine the frequency, hence it was defined in a dichotomous way. No interaction analysis between risk factors (contraceptives-thrombophilia, migraine-smoking, migraine-contraceptives, contraceptives-hypertension, etc) which may show a differential performance for stroke etiologies. Finally, National Institute of Health Stroke Scale (NIHSS) score wasn’t registered, nor was disability through modified Rankin score.

## Conclusions

This study shows a high frequency of traditional risk factors in young patients, which, being modifiable, highlights the need to optimize primary and secondary prevention plans. Additionally, a high frequency of illicit substance consumption such as cocaine and marihuana was observed. Infectious causes, such as tuberculous vasculitis, meningovascular syphilis, and neurocysticercosis related vasculitis, suggests a particular behavior in Latin-american countries, which must be considered in such patients. Undetermined causes require further studies.

## Supplementary Information

**Additional file 1: Supplementary Table 1.** Demographic data and risk factors according to sex and age.

## Data Availability

The datasets used and/or analyzed during the current study are available from the corresponding author on reasonable request.

## Abbreviations

AF: Atrial fibrillation
APS: Antiphospholipid syndrome
ASA: Atrial septal aneurysm
AT-III: Antithrombin III
CADASIL: Cerebral autosomal dominant arteriopathy with subcortical infarcts and leukoencephalopathy
CIOMS: Council for International Organizations of Medical Sciences
HIV: Human immunodeficiency virus
ICH: Intracranial hemorrhage
MRA: Magnetic resonance angiography
NIHSS: National Institute of Health Stroke Scale
PC: Protein C
PFO: Patent foramen ovale
PS: Protein S
SAH: Subarachnoid hemorrhage
SLE: Systemic lupus erythematosus
SVT: Sinus vein thrombosis
TOAST: Trial of Org 10,172 in Acute Stroke Treatment
TIA: Transitory ischemic attack
