# Supplementary material for: Ischemic stroke in young patients in Medellín, Colombia
Source: BMC Neurol. 2022 Sep 22;22:363. doi: 10.1186/s12883-022-02895-9 (PMC9494873; doi:10.1186/s12883-022-02895-9)
Supplement: Supplementary file 1 — Additional file 1: Supplementary Table 1. Demographic data and risk factors according to sex and age. [file 12883_2022_2895_MOESM1_ESM.docx]

**Supplementary material**

**Supplementary Table 1.** Demographic data and risk factors according to sex and age

|  | All (n=237) | Women (n=126) | Men (n=111) | *P* | Age 18 to 30 years (n=42) | Age 31 to 49 years (n=195) | *P* |
| --- | --- | --- | --- | --- | --- | --- | --- |
| Age, years | 40 (34-46) | 42 (34-46) | 39 (33-46) | 0.06 | 25.5 (23-28) | 43 (38-46) | * |
| Sex | - | 126 (53.2) | 111 (46.8) | * | 20F/22M | 106F/89M | 0.50 |
| Previous ischemic stroke | 30 (12.7) | 14 (11.1) | 16 (14.4) | 0.57 | 5 (11.9) | 25 (12.8) | 0.71 |
| Family history of ischemic stroke | 20 (8.4) | 11 (8.7) | 9 (8.1) | 0.97 | 2 (4.8) | 18 (9.2) | 0.63 |
| Obesity | 30 (12.7) | 21 (16.7) | 9 (8.1) | 0.06 | 4 (9.5) | 26 (13.3) | 0.79 |
| Dyslipidemia | 20 (8.4) | 10 (7.9) | 10 (9.0) | 0.30 | 0 | 20 (10.3) | 0.07 |
| Smoking | 70 (29.6) | 36 (28.6) | 34 (30.6) | 0.34 | 9 (21.4) | 61(31.3) | 0.49 |
| Arterial hypertension | 75 (31.7) | 45 (35.7) | 30 (27.0) | 0.13 | 3 (7.1) | 72 (36.9) | 0.00 |
| Diabetes | 19 (8.0) | 14 (11.1) | 5 (4.5) | 0.06 | 0 | 19 (9.7) | 0.08 |
| Sleep apnea | 0 | 0 | 0 | * | 0 | 0 | * |
| Ischemic cardiac disease | 7 (3.0) | 4 (3.2) | 3 (2.7) | 0.97 | 0 | 7 (3.6) | 0.29 |
| Heart failure | 14 (5.9) | 9 (7.1) | 5 (4.5) | 0.55 | 1 (2.4) | 13 (6.7) | 0.40 |
| Peripheral arterial disease | 3 (1.3) | 3 (2.4) | 0 (0.0) | 0.21 | 0 | 3 (1.5) | 0.52 |
| Valvular AF | 2 (0.8) | 2 (1.6) | 0 (0.0) | 0.33 | 0 | 2 (1.0) | 0.58 |
| Non valvular AF | 1 (0.4) | 0 (0.0) | 1 (0.9) | 0.44 | 0 | 1 (0.5) | 0.65 |
| Valve replacement | 12 (5.1) | 8 (6.3) | 4 (3.6) | 0.50 | 1 (2.4) | 11 (5.6) | 0.48 |
| PFO | 0 | 0 | 0 | * | 0 | 0 | * |
| ASA | 0 | 0 | 0 | * | 0 | 0 | * |
| Migraine | 22 (9.3) | 15 (11.9) | 7 (6.3) | 0.27 | 3 (7.1) | 19 (9.7) | 0.62 |
| APS | 2 (0.8) | 1 (0.8) | 1 (0.9) | 0.99 | 2 (4.8) | 0 | 0.01 |
| SLE | 8 (3.4) | 8 (6.3) | 0 (0.0) | 0.03 | 4 (9.5) | 4 (2.1) | 0.05 |
| Pregnancy / Postpartum | 2 (0.8) | 2 (1.6) | NA | * | 1 (2.4) | 1 (0.5) | 0.32 |
| Cancer | 7 (3.0) | 7 (5.6) | 0 (0.0) | 0.03 | 0 | 7 (3.6) | 0.33 |
| HIV | 2 (0.8) | 1 (0.8) | 1 (0.9) | 0.32 | 0 | 2 (1.0) | 0.65 |
| Syphilis | 3 (1.3) | 0 (0.0) | 3 (2.7) | 0.06 | 1 (2.4) | 2 (1.0) | 0.63 |
| Alcohol intake | 55 (23.2) | 10 (7.9) | 45 (40.5) | 0.00 | 11 (26.2) | 44 (22.6) | 0.59 |
| Cocaine | 23 (9.7) | 4 (3.2) | 19 (17.1) | 0.00 | 6 (14.3) | 17 (8.7) | 0.30 |
| Marihuana | 21 (8.9) | 5 (4.0) | 16 (14.4) | 0.02 | 6 (14.3) | 15 (7.7) | 0.24 |
| Other substances | 8 (3.4) | 1 (0.8) | 7 (6.3) | 0.06 | 3 (7.1) | 5 (2.6) | 0.18 |
| Use of oral contraceptives | 9 (3.8) | 9 (7.1) | NA | * | 1 (2.4) | 8 (4.1) | 0.55 |
| Low vitamin B12 levels | 27 (11.4) | 13 (10.3) | 14 (12.6) | 0.81 | 3 (7.1) | 24 (12.3) | 0.63 |
| Anticardiolipin antibodies | 17 (7.2) | 10 (7.9) | 7 (6.3) | 0.46 | 8 (19.0) | 9 (4.6) | 0.00 |
| Lupus anticoagulant | 18 (7.6) | 6 (4.8) | 12 (10.8) | 0.09 | 6 (14.3) | 12 (6.2) | 0.04 |
| PC deficiency | 3 (1.3) | 1 (0.8) | 2 (1.8) | 0.33 | 1 (2.4) | 2 (1.0) | 0.15 |
| PS deficiency | 7 (3.0) | 3 (2.4) | 4 (3.6) | 0.26 | 0 | 7 (3.6) | 0.01 |
| Hyperhomocysteinemia | 4 (1.7) | 2 (1.6) | 2 (1.8) | 0.72 | 0 | 4 (2.1) | 0.63 |
| Factor V Leiden | 1 (0.4) | 0 | 1 (0.9) | 0.32 | 0 | 1 (0.5) | 0.87 |
| Prothrombin deficiency | 0 | 0 | 0 | * | 0 | 0 | * |
| Other thrombophilia:  AT-III deficiency | 1 (0.4) | 0 | 1 (0.9) | 0.34 | 0 | 1 (0.5) | 0.46 |

Data expressed as median (interquartile range) or n (%)
*P* value is from Chi square test, but if expected value < 5, is from Fisher’s test.
* non-logic comparison
M: Male / F: Female

AT-III: antithrombin III; ASA: atrial septal aneurysm; AF: Atrial fibrillation; PFO: Patent foramen ovale; SLE: systemic lupus erythematosus; PC: protein C; PS: protein S; APS: antiphospholipid syndrome; HIV: human immunodeficiency virus; NA: does not apply.
